# Global analysis of H3K27me3 as an epigenetic marker in prostate cancer progression

**DOI:** 10.1186/s12885-017-3256-y

**Published:** 2017-04-12

**Authors:** Marjolaine Ngollo, Andre Lebert, Marine Daures, Gaelle Judes, Khaldoun Rifai, Lucas Dubois, Jean-Louis Kemeny, Frederique Penault-Llorca, Yves-Jean Bignon, Laurent Guy, Dominique Bernard-Gallon

**Affiliations:** 1 Department of Oncogenetics, Centre Jean Perrin – CBRV, 28 place Henri Dunant, BP 38, 63001 Clermont-Ferrand, France; 2 INSERM U 1240, IMOST, 58 rue Montalembert-BP184, 63005 Clermont-Ferrand, France; 3 University Blaise Pascal, Institut Pascal UMR 6602 CNRS/UBP, 24 Avenue des Landais, Aubière, France; 4 Department of Biopathology, Gabriel Montpied Hospital, 58 rue Montalembert, 63000 Clermont-Ferrand, France; 5 Department of Biopathology, Centre Jean Perrin, 58 rue Montalembert, 63000 Clermont-Ferrand, France; 6 Department of Urology, Gabriel Montpied Hospital, 58 rue Montalembert, 63000 Clermont-Ferrand, France

**Keywords:** ChIP-on-chip, Epigenetics, Histone methylation, H3K27me3, Prostate, Cancer

## Abstract

**Background:**

H3K27me3 histone marks shape the inhibition of gene transcription. In prostate cancer, the deregulation of H3K27me3 marks might play a role in prostate tumor progression.

**Methods:**

We investigated genome-wide H3K27me3 histone methylation profile using chromatin immunoprecipitation (ChIP) and 2X400K promoter microarrays to identify differentially-enriched regions in biopsy samples from prostate cancer patients. H3K27me3 marks were assessed in 34 prostate tumors: 11 with Gleason score > 7 (GS > 7), 10 with Gleason score ≤ 7 (GS ≤ 7), and 13 morphologically normal prostate samples.

**Results:**

Here, H3K27me3 profiling identified an average of 386 enriched-genes on promoter regions in healthy control group versus 545 genes in GS ≤ 7 and 748 genes in GS > 7 group. We then ran a factorial discriminant analysis (FDA) and compared the enriched genes in prostate-tumor biopsies and normal biopsies using ANOVA to identify significantly differentially-enriched genes. The analysis identified *ALG5*, *EXOSC8*, *CBX1*, *GRID2*, *GRIN3B*, *ING3*, *MYO1D*, *NPHP3*-*AS1*, *MSH6*, *FBXO11*, *SND1*, *SPATS2*, *TENM4* and *TRA2A* genes. These genes are possibly associated with prostate cancer. Notably, the H3K27me3 histone mark emerged as a novel regulatory mechanism in poor-prognosis prostate cancer.

**Conclusions:**

Our findings point to epigenetic mark H3K27me3 as an important event in prostate carcinogenesis and progression. The results reported here provide new molecular insights into the pathogenesis of prostate cancer.

## Background

Epigenetic alterations play a critical role in cancer initiation and progression in addition to genetic alterations. Epigenetics includes changes such as DNA methylation, microRNA and histone modifications, which together make up the epigenome [1]. Here we focused on the histone modifications that play a crucial role in cancer development and progression. Chromatin structure plays an important role in regulating various nuclear functions such as transcription, replication, recombination and DNA repair. Regulation of gene expression is known to be involved in the binding of transcription factors in target gene promoters but it is also dependent on how the epigenetic events, including histone marks, are characterized. Basically, the local modification of chromatin structure by histone modifications can lead to activation or inactivation of gene expression. For example, some histone modifications such as tri-methylated histone H3 at lysine 27 (H3K27me3) are known to inactive gene expression.

Studies of alteration in histone methylation at whole-genome scale bring insight into gene regulation. Global changes in histone H3 are emerging as a new biomarker in malignant transformation [2, 3]. Likewise, histone-modifier enzymes control dynamic transcription of gene expression in normal and cancer cells, enabling key physiopathological processes to take place [4, 5]. Polycomb-group proteins (PcGs) are involved in silencing gene expression, particularly during development and differentiation stages [6, 7], and also play the major role in nuclear reprogramming and chromatin remodeling [8]. PcGs are organized into two main polycomb-repressive complexes (PRCs), PRC1 and PRC2, that control gene silencing through post-translational histone modifications [9, 10].

At specific loci, the histone methyltransferase enhancer of zeste homolog 2 (EZH2), a subunit of PRC2, catalyzes H3K27 trimethylation, leading to chromatin compaction and subsequently silencing of genes in prostate cancer [11]. Abnormal functions of PcGs are one of the main factors involved in the initiation and progression phases in many cancers, including prostate cancer [12]. EZH2 is highly overexpressed in prostate cancer and strongly associated with epigenetic silencing in cancer. EZH2 is so prominently involved in aggressive cell growth, metastasis, drug resistance and stem cell maintenance that it has become an attractive therapeutic target in prostate cancer [13–15]. Previous studies show that EZH2 up-regulation is correlated with H3K27me3 deregulation and poor-prognosis prostate tumor [16]. The H3K27me3 repressive mark has been found on many gene promoters that are silenced [17], and genome-wide profiling studies of the H3K27me3 mark in metastatic and prostate cancer cells suggest a silencing function of EZH2 in prostate cancer [18, 19].

This study used 34 human prostate biopsies and chromatin immunoprecipitation (ChIP) assays to investigate the interactions between H3K27me3 and gene promoter in prostate cancer. ChIP coupled with promoter microarrays enabled us to determine the entire spectrum of in vivo DNA binding sites of the H3K27me3 repressive mark. Identifying region-specific H3K27me3 patterns also helps address additional questions, such as how these observations may resolve or at least suggest causal relationships between histone methylation and prostate cancer progression.

We demonstrated that average number of H3K27me3-enriched genes was higher in tumor tissues than normal tissues. Then, after factorial discriminant analysis and ANOVA, we characterized the significant interaction of H3K27me3 with *ALG5*, *EXOSC8*, *CBX1*, *GRID2*, *GRIN3B*, *ING3*, *MYO1D*, *NPHP3*-*AS1*, *MSH6*, *FBXO11*, *SND1*, *SPATS2*, *TENM4* and *TRA2A* in tumor tissues compared to normal tissues. These genes were all more H3K27me3-enriched and were able to discriminate different groups according to Gleason score.

## Methods

### Prostate patient samples

Normal and tumoral prostate biopsies were obtained from 34 patients (Table 1) diagnosed with prostate cancer at Clermont-Ferrand University Hospital (France). All biopsies were kept in nitrogen. A pathologist performed tumor evaluation. Patients did not receive chemotherapy before clinical examination. All subjects gave written informed consent to the study, which was approved by the French Ministry for Higher Education and Research (DC-2008-558).

**Table 1:** Clinical and biological characteristics of patients

Cases	TT	NT
Total cases (*n* = 34)	21	13
Age at diagnosis (years)		
< 49	0	0
50–59	2	2
60–69	11	8
> 70	8	3
Baseline PSA (ng/mL)		
< 4	0	0
4–10	8	9
10–20	8	4
> 20	5	0
Clinical stage		
T1c	7	-
T2	8	-
T3	6	-
Gleason Score		
≤ 7	10	-
> 7	11	-

*TT* tumoral tissues, *NT* normal tissues, *PSA* prostate-specific antigen

### Chromatin immunoprecipitation (ChIP)

Tissues were fixed for 15 min at room temperature (RT) using 1% formaldehyde in phosphate buffered saline (PBS) containing protease inhibitors. Reversal of crosslinking was performed by incubation with 0.125 M glycine for 5 min at RT. Each pellet was re-suspended with lysis buffer (5 mM PIPES pH 8, 0.85 mM KCL, 0.5% Igepal) supplemented with 1X protease inhibitor cocktail, and sonicated for 30 min in 30 s ON/30 s OFF cycles (Bioruptor, Diagenode). The lysate was centrifuged at 14,000 *g* for 10 min and the supernatant transferred to a fresh tube. Optimal fragmentation was achieved by testing various sonication conditions on chromatin followed by DNA isolation and gel electrophoresis estimation of sonication efficiency. ChIP was performed using an AutoTrue MicroChIP kit (Diagenode #C01010140) on a SX-8G IP-Star Compact Automated System (Diagenode) as per the manufacturer’s instructions. Immunoprecipitation was performed using 3 μg of anti-H3K27me3 (Diagenode #C15410195) and non-specific IgG (Diagenode). Reverse crosslinking was performed with 5 M NaCl for 4 h at 65 °C. The immunoprecipitated DNA and input samples were purified using MicroChIP DiaPure columns (Diagenode #C03040001) and eluted with TE buffer. After ChIP, the crosslink was reversed and the DNAs were purified. To assess the quality and efficiency of the ChIP procedure, quantitative PCR was performed to assess the enrichment of known target genes. *GAPDH*, a housekeeping gene, was used as negative control for H3K27me3 ChIP. *TSH2B* gene, which is present in heterochromatin, was used as positive control for H3K27me3. *TSH2B* showed strong enrichment of H3K27me3 while *GAPDH* gene showed weak enrichment. Only samples with an enrichment of H3K27me3 above 5 were selected for ChIP-on-Chip analyses. Quantitative PCR was performed using SYBR Green Mix (Applied Biosystems #4309155) following the manufacturer’s instructions. The samples were amplified using an Applied Biosystems ABI Prism®7900 HT Real-Time PCR System (Applied Biosystems). PCR program was 95 °C for 3 min and 40 cycles of 95 °C for 30 s, 60 °C for 30 s and 72 °C for 30 s. The IP and input DNA were then subjected to microarray hybridization.

### Promoter microarray hybridization

After ChIP, the immunoprecipitated DNA was pre-amplified with a whole-genome amplification kit (Sigma #WGA2) following the manufacturer’s protocol, and 2 μg DNA was labeled using a SureTag complete DNA labeling kit (Agilent Technologies #51904240) at 37 °C for 2 h then 65 °C for 10 min. Input DNA was then labeled with cyanine 3 while immunoprecipitated DNA was labeled with cyanine 5. Both samples were purified on columns and eluted in TE buffer. Labeled DNAs were mixed and competitively hybridized to DNA microarrays. Hybridization was carried out on 2X400K Sure Print G3 Human promoter microarrays (Agilent #G4874A) in the presence of human Cot-1 DNA for 40 h at 65 °C and the slides were washed according to Agilent’s procedure. After washing, the slides were scanned using an Agilent microarray scanner, and intensity of fluorescent signals was extracted using Agilent feature extraction 11.2 software.

Each slide contained two identical arrays and each microarray contained 414,043 (60-mer) oligonucleotide probes spaced every 172 bp across promoter regions including −5.5 Kb upstream and +2.5 Kb downstream of identified transcriptional start sites (TSS). The probes covered 21,000 of the best-defined human transcripts represented as RefSeq genes.

### Data analysis

For the H3K27me3-enriched gene analysis between normal tissues and tumors, ChIP-on-chip data were processed using RINGO software 1.26.1, then the microarray data was analyzed on R software using several Bioconductor packages (www.bioconductor.org/). Enriched regions were defined from enriched probes using the criteria of at least 3 enriched probes within the region. For each probe on the array, a score was calculated as follows: score = ∑ (enrichment values Probes-Enrichment Threshold). Only genes with a threshold of >1.5 were considered as differently enriched. Genes with a score of least than 1.5 were removed from analysis, as were genes with missing data in more than 30% of the samples.

### Gene annotation

Gene annotation was carried out using the ENSEMBL annotation system. We generated enrichment profiles for H3K27me3 in tumor samples compared to normal tissues. After determining the enriched regions for H3K27me3 modifications, RefSeq genes were downloaded from the ENSEMBL database.

### Statistical analysis

H3K27me3 sites were defined as differentially enriched if the Enrichment Score was >1.5, and for each H3K27me3 site the mean Enrichment Score level was compared in tumor tissue group versus normal tissue. Factorial discriminant analysis (FDA) and ANOVA were performed to discriminate the three groups. Data was analyzed using R statistics. Thresholds set for statistical significance were *p < 0.05 and **p < 0.01.

## Results

### Whole-genome screening of H3K27me3 epigenetic marks in human prostate cancer

In order to grasp the role H3K27me3 marks in prostate cancer progression in 34 patients, we investigated H3K27me3 mark binding to determine whether it correlates with tumor progression. First, to examine the epigenetic signature of H3K27me3 in prostate cancer, we mapped the global promoter occupancy profile of H3K27me3 in prostate cancer compared to normal biopsies using ChIP-on-chip methods.

Samples were divided into three groups: 13 normal prostate tissues, 11 prostate cancer tissues with Gleason score > 7 and 10 prostate cancer tissues with Gleason score ≤ 7. Enriched regions were defined via an Enrichment Score (ES)-based sliding window approach using RINGO software [20], then the binding sites were annotated to human genes using the ENSEMBL database. Importantly, we selected genes whose enrichment score was greater than 1.5 in the promoter regions. Among the 21,000 genes analyzed in human 2X400K, we calculated the average of H3K27me3 modifications among patients in each group. We identified an average of 386 genes with H3K27me3 marks in the promoter regions in healthy control group versus 545 genes in GS ≤ 7 and 748 genes in GS > 7 group. These results suggested that there are more extensive H3K27me3-enriched gene promoters in advanced disease than normal tissues.

**Fig. 1:**
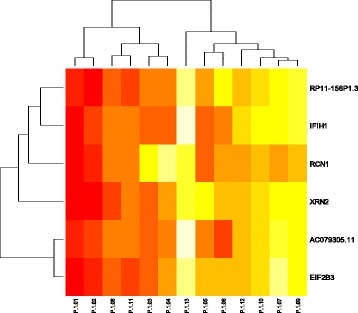
Hierarchical clustering on a set of 13 normal biopsies. The scaled enrichment score of individual patients is plotted in a *red-yellow* scale. Color intensity reflects magnitude of enrichment score, with red indicating high H3K27me3 enrichment and *yellow* indicating low H3K27me3 enrichment. Columns represent individual tissues. Rows represent the genes. The dendogram represents overall similarities in patient profiles

**Fig. 2:**
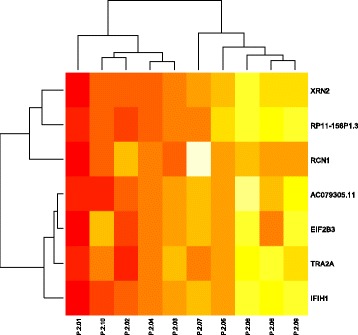
Hierarchical clustering analysis of tumor tissues with Gleason score ≤ 7. The scaled enrichment score of individual patients is plotted in a *red-yellow* scale. Color intensity reflects magnitude of enrichment score, with red indicating high H3K27me3 enrichment and yellow indicating low H3K27me3 enrichment. Columns represent individual tissues and rows represent the genes. The dendogram represents overall similarities in patient profiles

To list enriched-genes, we performed a hierarchic clustering analysis that also helped to see similarities between patients. The genome-scale H3K27me3 profile of each group was then compared. Figure 1 showed the enrichment of genes such *as IFIH1, RCN1, XRN2, EIF2B, RP11-156P1.3* and *AC079305.11*. However, differences at the genetic and molecular level could explain by the interindividual difference in control group. Interestingly, all of these genes are shared with all patients in GS ≤ 7 group. Despite interindividual variability, we identified one gene that is specific to GS ≤ 7 group, *TRA2A* gene (Fig. 2).

**Fig. 3:**
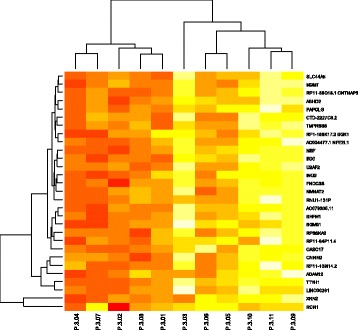
Hierarchical clustering analyses of patients with Gleason score > 7. The scaled enrichment score of individual patients is plotted in a *red-yellow* scale. Color intensity reflects magnitude of enrichment score, with red indicating high H3K27me3 enrichment and *yellow* indicating low H3K27me3 enrichment. Columns represent individual tissues. Rows represent the genes. The dendogram represents overall similarities in patient profiles

The greatest changes occurred in GS > 7 group where we observed several H3K27me3-enriched genes such as *MGMT*, *SLC4A4*, *ABHD2*, *PAPOLG, NSF, ING3*, *TMPRSS6, FNDC3B* (Fig. 3).

### H3K27me3 epigenetic marks correlate with prostate cancer aggressiveness

**Fig. 4:**
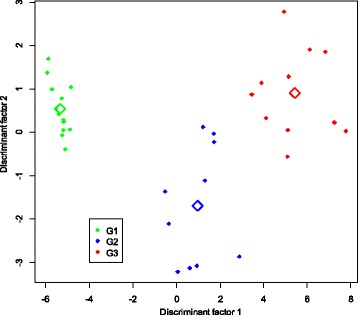
Factorial discriminant analysis (FDA) of microarray-based genome-wide H3K27me3 profiles derived from prostate biopsies. Prostate biopsies were obtained from healthy patients (*n* = 13), prostate cancer patients with Gleason score ≤ 7 (*n* = 10) and prostate cancer patients with Gleason score > 7 (*n* = 11). Data showed a well-defined separation between patients according to Gleason score and H3K27me3 markers. Center of gravity for each group is reported as the empty symbol. G1, healthy group; G2, prostate cancer with Gleason score ≤ 7; G3 prostate cancer with Gleason score > 7

Discriminant analysis on the whole microarray dataset showed a clear segregation of samples with GS ≤ 7, GS > 7 and healthy controls. This analysis indicated that H3K27me3 profiles could classify prostate cancer patients (Fig. 4).

### Enriched genes with H3K27me3 marks in prostate cancer tissues versus normal tissues

**Fig. 5:**
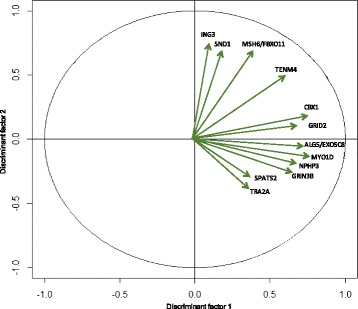
Factorial discriminant analysis. The results represent differentially H3K27me3-enriched genes between prostate cancer tissues versus normal tissues. The Highly enrichment correlated with GS > 7

Even though the global pattern of enriched genes in carcinoma tissues and normal tissues showed variability between patients, it was possible to identify differentially H3K27me3-enriched genes involved in prostate cancer based on enrichment score level. Using ANOVA, we identified genes that were significantly enriched between both tumor groups versus normal samples. The significance of the enrichment score values of *ALG5*, *EXOSC8*, *CBX1*, *GRID2*, *GRIN3B*, *ING3*, *MYO1D*, *NPHP3-AS1*, *MSH6*, *FBXO11*, *SND1*, *SPATS2*, *TENM4 and TRA2A* genes are shown in Table 2. Association of H3K27me3 enrichment and clinico-pathological variables like stage and PSA level did not show any significance. However, only Gleason score correlated with the H3K27me3 enrichment on genes (Fig. 5).

**Table 2:** Compiled statistics of FDA and ANOVA results

Gene name	Coordinate axis 1	Coordinate axis 2	*p* value	Significance
*ALG5/EXOSC8*	0.815	−0.154	0.001	**
*CBX1*	0.643	0.079	0.038	*
*GRID2*	0.655	0.056	0.034	*
*GRIN3B*	0.627	−0.168	0.039	*
*ING3*	0.074	0.748	0.020	*
*MYO1D*	0.666	−0.168	0.023	*
*NPHP3-AS1*	0.640	−0.186	0.031	*
*MSH6/FBXO11*	0.366	0.561	0.047	*
*SND1*	0.187	0.653	0.049	*
*SPATS2*	0.579	−0.352	0.031	*
*TENM4*	0.592	0.320	0.032	*
*TRA2A*	0.563	−0.412	0.025	*

Coordinate axes refer to FDA data values (Fig. 4). *P* value refers to ANOVA data. *<0.05 **< 0.01

Differentially H3K27me3-enriched genes in prostate cancer tissues compared to normal biopsies

## Discussion

There is a pressing need for further work on the molecular mechanisms underlying prostate cancer in order to improve prognosis, diagnosis and treatment. In particular, characterizing the functional role of genetics in prostate cancer by observing the new target gene would help identify potential drugs. Here we report a set of target genes that interact with H3K27me3 in prostate cancer.

Comparison of the H3K27me3 profiles of prostate cancer tissues versus normal tissues revealed an average of 386 enriched-genes on promoter regions in healthy control group versus 545 genes in GS ≤ 7 and 748 genes in GS > 7 group. These data characterize H3K27me3 as an epigenetic feature of histone methylation-related prostate cancer progression. For the study design and criteria used here, patients were pooled at every stage analyzed to reduce the number of non-common genes. This pooling brought together individuals showing the lowest disparity of results in each group. The goal was to identify common genes that may be significant and representative of any disease stage.

Among the genes identified as being differentially regulated by H3K27me3 were *TRA2A, FBXO11, ING3* and *CBX1*.

Note that *TRA2A* gene was shown to discriminate control group and GS ≤ 7 group. *TRA2A* plays a role in the regulation of pre-mRNA splicing after phosphorylation and binding to specific RNA [21, 22]. This gene has not been studied yet in connection with prostate tumorigenesis. Furthermore, *TRA2A* appeared to be H3K27me3-enriched in all patients and could thus serve as an epigenetic marker for early prostate cancer screening. In contrast, GS > 7 patients showed high H3K27me3 enrichment at the *TRA2A* promoter compared to both the GS ≤ 7 and normal groups, suggesting its major role in prostate cancer.

*FBXO11*, *ING3* and *CBX1* genes all play a role in epigenetic control and regulation of chromatin. *FOBXO11* is an arginine methyltransferase that symmetrically dimethylates arginine residues. A recent study in epithelial cancer demonstrated cells that FBXO11 induced an increase of Snai1 and a decrease of E-cadherin to prevent tumor progression, thus characterizing FBXO11 as a tumor suppressor [23]. H3K27me3 enrichment on the *FBXO11* promoter may mediate the repression of this gene in prostate cancer.

The other tumor suppressor gene characterized here was *ING3*. This inhibitor of growth-family protein was initially identified as a tumor suppressor with altered regulation in a variety of cancer types, including in colorectal cancer cells [24, 25]. ING is, however, a protein involved in chromatin remodeling. In fact, ING3 acts as a reader of epigenetic code through specific recognition of H3K4me3 and can affect HAT and HDAC activity by serving as members of the Sin3A, Tip60 or Moz/Morf HAT complexes [26]. Our results showed the epigenetic regulation of ING3 via H3K27me3 in prostate cancer suggesting putative tumor suppressor gene silencing by histone methylation in prostate cancer. These data suggest that the ING3 locus may locate in bivalent domains marked by both H3K27me3 and H3K4me3 and that ING3 may thus play a critical role in cancer development.

*CBX1* (*HP1α*) is a member of the heterochromatin protein 1 family (HP1s) that plays a role in the formation and maintenance of heterochromatin. This gene encodes a non-histone protein that is able to bind to histone proteins via methylated lysine residues. Genome-wide localization analysis reveals H3K27me3 binding at CBX1 promoter regions and thus points to heterochromatin formation corresponding to gene silencing in prostate cancer. It has already been shown that CBX1 is downregulated in invasive breast cancer cells [27], and our findings show that a novel epigenetic mechanism might involve CBX1 in transcriptional regulation, thus providing new insight for further elucidation of the molecular mechanisms causing the *CBX1* downregulation in cancer cells.

Other genes found to be H3K27me3-enriched in prostate cancer tissues compared to normal tissues include *MYO1D*, *TENM4*, *GRIN3B*, all of which are involved in cell communication and cell adhesion [28–30]. These genes have not yet been described in human cancer, but disrupted intracellular adhesion is a prerequisite for tumor cell invasion and metastasis.

Furthermore, *MSH6* gene was found to be epigenetically regulated in prostate cancer. *MSH6* is DNA mismatch repair genes. The loss-of-function DNA mismatch repair genes are linked to mutation or epigenetic silencing [31]. In addition, the hypermutated subtype of prostate cancer is chiefly due to loss-of-function mutations in MSH6 in advanced prostate cancer [32]. We thus hypothesized that transcriptionally-repressed *MSH6* gene might be related to H3K27me3 epigenetic modification in prostate cancer.

The comparison of normal and tumor prostate samples revealed far more H3K27me3 marks in advanced tumor tissues compared to normal tissues. These alterations could have major impacts on global gene expression via chromatin state. Our observations suggested that H3K27me3 marks are active in tumor tissues. Increased H3K27me3 marks could be explained by the activity of the PcG such as EZH2*,* which is frequently over-expressed in prostate cancer [13, 33]. These results implied that the most numerous epigenetic changes from normal tissues to prostate cancer tissues were gains of H3K27me3 marks.

Although the results reported here cannot confirm a repressor status on the increase of H3K27me3 marks on genes, they can serve to formulate a hypothesis. Performing qPCR to validate the selected differentially-enriched genes would help gauge the reliability of the ChIP-on-chip data reported here. Chromatin accessibility could be analyzed by ChIP-qPCR with RNA polymerase II. A previous study had shown that combinations of histone marks, for example gain of H3K4me3 and loss of H3K27me3 or gain of H3K27me3 and loss of H3K4me3, were strongly associated with up-regulated and down-regulated genes in prostate cancer cells. Nevertheless, gain or loss of just one mark is unlikely to prove sufficient for transcriptional changes [34].

## Conclusions

The findings of this study provide key insight for elucidating the regulation of epigenetic changes in prostate cancer. We demonstrated that global H3K27me3 histone modifications correlated with Gleason score in prostate cancer. A set of epigenetic markers was identified, and the data suggests a complex interplay between EZH2 and H3K27me3 histone modifications.

## Abbreviations

ChIP: Chromatin immunoprecipitation
EZH2: Enhancer of zeste homolog 2
FDA: Factorial discriminant analysis
GS: Gleason score
H3K27me3: Histone 3 lysine 27 trimethylation
H3K4me3: Histone 4 lysine 4 trimethylation
HAT: Histone acetyltransferase
HDAC: Histone deacetylase
HP1: Heterochromatin protein 1 family
PBS: Phosphate buffered saline
PcGs: Polycomb-group proteins
PRC: Polycomb-repressive complex
TSS: Transcriptional start sites
